# Supplementary material for: Antibacterial Application on Staphylococcus aureus Using Antibiotic Agent/Zinc Oxide Nanorod Arrays/Polyethylethylketone Composite Samples
Source: Nanomaterials (Basel). 2019 May 8;9(5):713. doi: 10.3390/nano9050713 (PMC6566776; doi:10.3390/nano9050713)
Supplement: Supplementary file 1 [file nanomaterials-09-00713-s001.pdf]

Supporting Information

Antibacterial Application on *Staphylococcus aureus* Using Antibiotic Agent/Zinc Oxide Nanorod Arrays/Polyethylethylketone Composite Samples

Dave W. Chen [1,2,*], Kuan-Yi Lee [1,3], Min-Hua Tsai [1,3], Tung-Yi Lin [1,2], Chien-Hao Chen [1,2] and Kong-Wei Cheng [1,3,*]

1 Department of Orthopaedic Surgery, Chang Gung Memorial Hospital, Keelung Branch, Taoyuan, Taiwan; m0423042@stmail.cgu.edu.tw (K.-Y.L.); m0523048@stmail.cgu.edu.tw (M.-H.T.); ross_1222@hotmail.com (T.-Y.L.); chchen1982@gmail.com (C.-H.C.)

2 College of Medicine, Chang Gung University, Taoyuan, Taiwan

3 Department of Chemical and Materials Engineering, Chang Gung University, Taoyuan, Taiwan

* Correspondence: mr5181@adm.cgmh.org.tw (D.W.C); kwcheng@mail.cgu.edu.tw (K.-W.C)

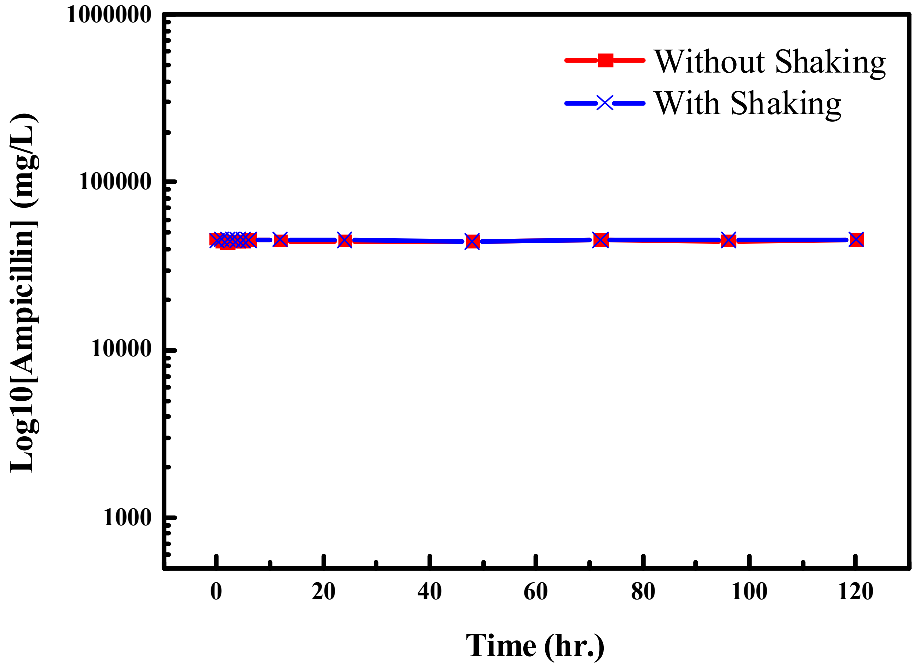

(a)

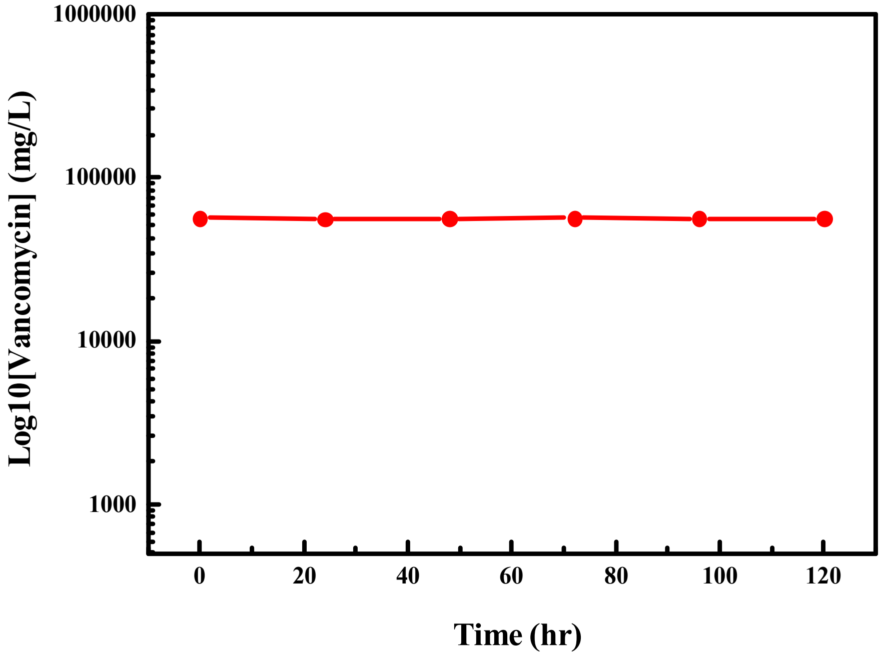

(b)

Figure S1 The variations of concentration for (a) ampicillin and (b) vancomycin in the solution bath containing PEEK samples as a function of time.

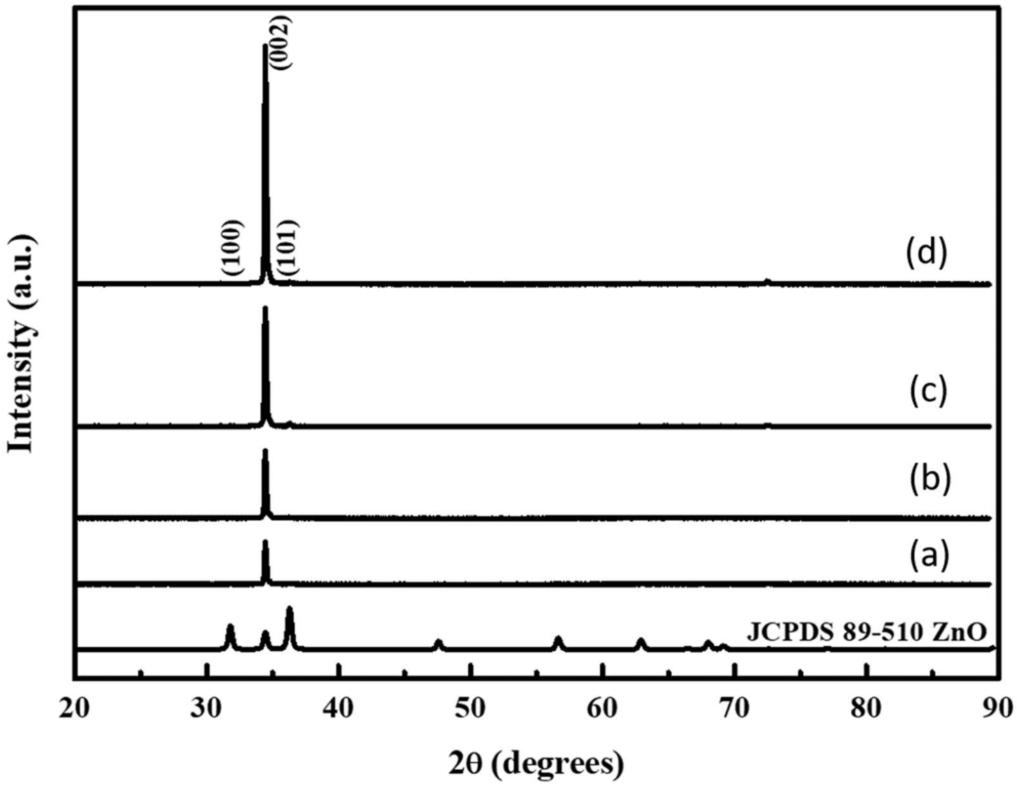

Figure S2 XRD patterns of ZnO samples grown on the glass substrates with various ammonium hydroxide concentrations in the precursor solution baths. (a) 4M, (b) 4.5M, (c) 5M and (d) 5.5 M for ammonium hydroxide in reaction solution, respectively.

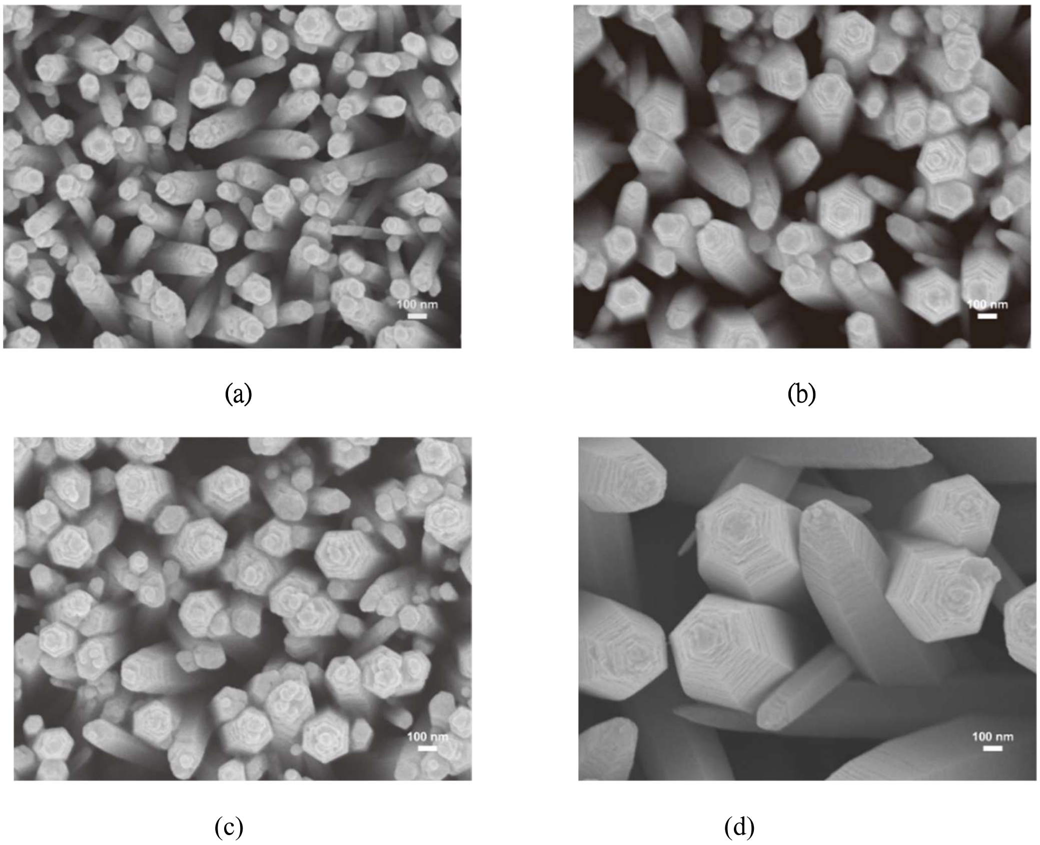

Figure S3 SEM images of ZnO samples on the glass substrate with various ammonium hydroxide concentrations in the precursor solution baths. (a) 4M, (b) 4.5M, (c) 5M and (d) 5.5 M for ammonium hydroxide in reaction solution, respectively.

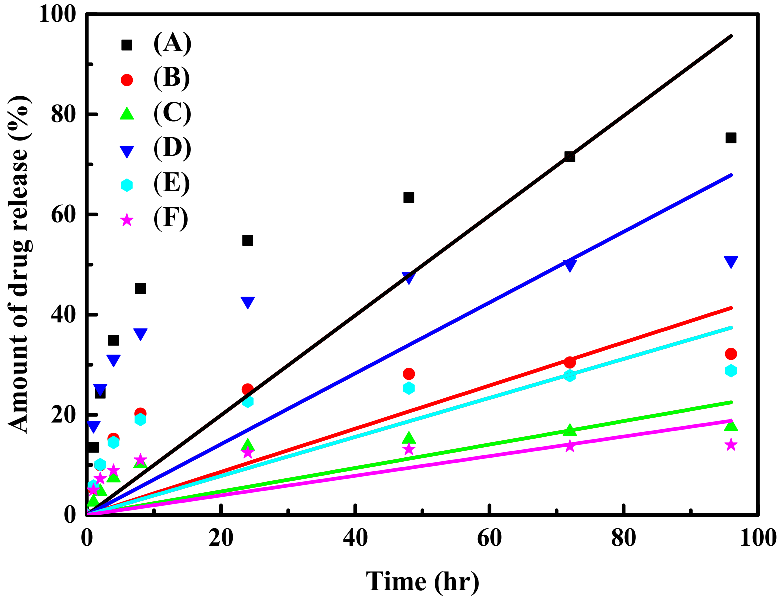

100
80
60
40
20
0
Amount of drug release (%)
(A)
(B)
(C)
(D)
(E)
(F)
0
20
40
60
80
100
Time (hr)

(a)

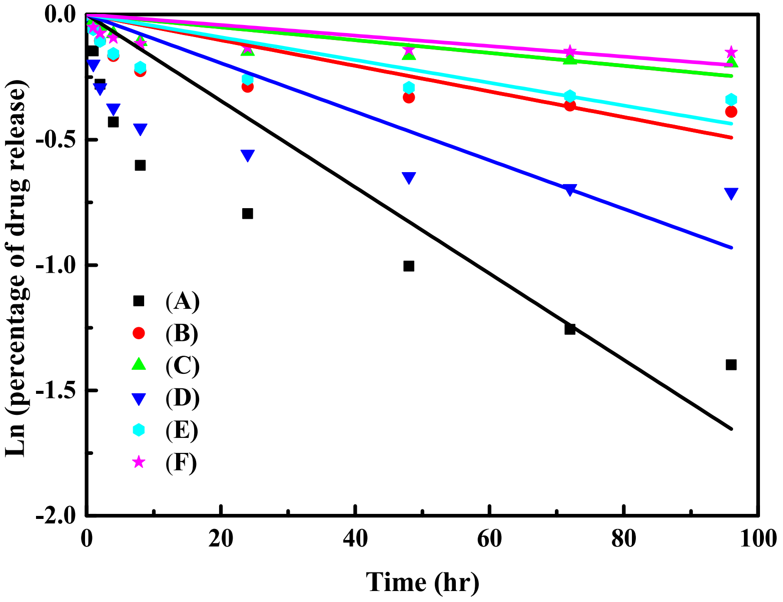

0.0
-0.5
-1.0
-1.5
-2.0
Ln (percentage of drug release)
(A)
(B)
(C)
(D)
(E)
(F)
0
20
40
60
80
100
Time (hr)

(b)

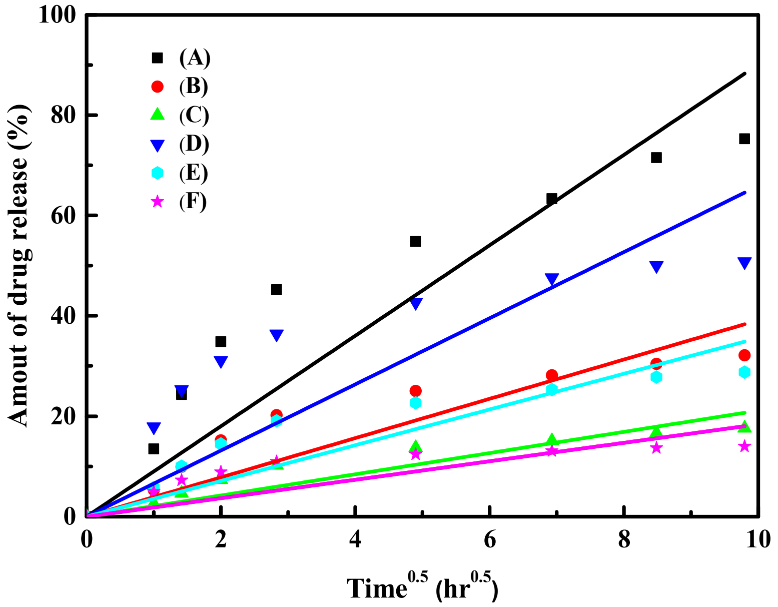

(c)

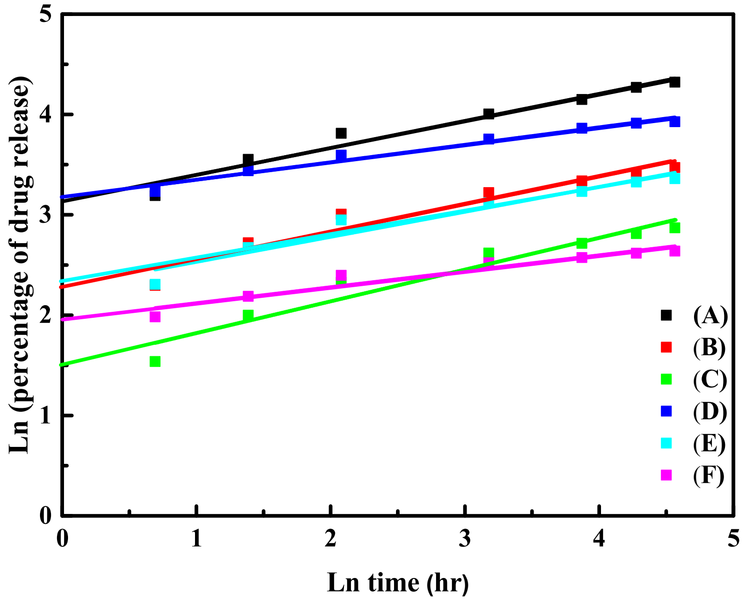

(d)

Figure S4 Fitting results of (a) zero-order, (b) first order, (c) Higuch and (d) Koresmeyer-Pappas kinetic models for samples (A)-(F) in the buffer solutions.
